# A Review of Molecular Responses of Catfish to Bacterial Diseases and Abiotic Stresses

**DOI:** 10.3389/fphys.2018.01113

**Published:** 2018-08-23

**Authors:** Tao Zhou, Zihao Yuan, Suxu Tan, Yulin Jin, Yujia Yang, Huitong Shi, Wenwen Wang, Donghong Niu, Lei Gao, Wansheng Jiang, Dongya Gao, Zhanjiang Liu

**Affiliations:** ^1^The Fish Molecular Genetics and Biotechnology Laboratory, Aquatic Genomics Unit, School of Fisheries, Aquaculture and Aquatic Sciences, Auburn University, Auburn, AL, United States; ^2^Department of Biology, College of Art and Sciences, Syracuse University, Syracuse, NY, United States

**Keywords:** enteric septicemia of catfish, columnaris, *Aeromonas*, heat, hypoxia, disease, stress, quantitative trait locus

## Abstract

Catfish is one of the major aquaculture species in the United States. However, the catfish industry is threatened by several bacterial diseases such as enteric septicemia of catfish (ESC), columnaris disease and *Aeromonas* disease, as well as by abiotic stresses such as high temperature and low oxygen. Research has been conducted for several decades to understand the host responses to these diseases and abiotic stresses. With the development of sequencing technologies, and the application of genome-wide association studies in aquaculture species, significant progress has been made. This review article summarizes recent progress in understanding the molecular responses of catfish after bacterial infection and stress challenges, and in understanding of genomic and genetic basis for disease resistance and stress tolerance.

## Introduction

Diseases cause significant amount of economic losses in aquaculture (Meyer, 1991). The disease problems in aquaculture systems are increased following exposure to abiotic stresses. Thus, diseases and abiotic stresses are related, and they together post greater challenges for aquaculture industry. Great efforts have been made to elucidate the molecular mechanisms for disease resistance and stress tolerance. A good example is the identification of quantitative trait locus (QTL) and determination of the causal gene for the infectious pancreatic necrosis virus (IPNV) resistance in Atlantic salmon *Salmo salar* (Houston et al., 2008, 2010; Moen et al., 2015), and such information is now applied in the aquaculture industry to control IPVN. As summarized in a recent white paper (Abdelrahman et al., 2017), increasingly more QTL studies are being conducted to identify genetic variants and genomic regions associated with disease resistance and stress tolerance in various aquaculture species, including channel catfish (*Ictalurus punctatus*), Atlantic salmon, rainbow trout (*Oncorhynchus mykiss*), Asian seabass (*Lates calcarifer*), and Japanese flounder (*Paralichthys olivaceus*). Moreover, transcriptomic analyses after disease infection and stress challenges were conducted in aquaculture species (Qian et al., 2014; Eissa and Wang, 2016), providing insights into differentially expressed genes, and their involved pathways, all providing information for the development of genome-based technologies for disease control for a sustainable aquaculture industry.

Catfish is a diverse group with approximately 4,000 species. Several catfish species are important for aquaculture across the world including African catfish (*Clarias gariepinus*), walking catfish (*Clarias batrachus*), shark catfish (*Pangasius bocourti*), Thai catfish (*Clarias macrobrachium*), channel catfish and blue catfish (*I. furcatus*). In North America, channel catfish and hybrid catfish produced by mating female channel catfish with male blue catfish are the most important. Catfish production is the largest of U.S. finfish aquaculture, valued at approximately $423 million in 2012, and the economic impact of catfish industry is $2.5 billion (Dunham and Elaswad, 2018). However, the catfish production declined in recent years due to various reasons including fierce international competition, increased costs of fish feed, and disease problems. Bacterial diseases cause major problems for the U.S. catfish industry. In particular, three bacterial diseases including enteric septicemia of catfish (ESC), columnaris disease, and motile *Aeromonas* septicemia (MAS) are the primary disease concerns (Wagner et al., 2002; Plumb and Hanson, 2011). In addition to disease problems, abiotic stresses such as high water temperature and low levels of dissolved oxygen also lead to significant economic losses (Burggren and Cameron, 1980; Ficke, 2005; Welker et al., 2007).

Huge efforts have been devoted to reducing the economic losses including development of vaccines, and generation of disease resistant and stress tolerant catfish strains and families. For the later, great genetic variations among catfish species and strains in response to diseases and stresses provide biological sources for the analysis of genetic and epigenetic regulation of disease resistance. After the publication of the channel catfish reference genome sequence (Liu et al., 2016), rapid progress has been made for the determination of QTL associated with disease resistance (Geng et al., 2015; Zhou et al., 2017b; Shi et al., 2018; Tan et al., 2018) and stress tolerance (Jin et al., 2017; Wang et al., 2017b; Zhong et al., 2017).

In this paper, we review progress made in understanding pathogenesis, host responses, and genomic basis for disease resistance of ESC, columnaris, and *Aeromonas* diseases, as well as for abiotic stress such as heat stress, and hypoxia.

## Part I: Enteric Septicemia of Catfish (Esc)

### Impact of ESC Disease to the Catfish Industry

Enteric septicemia of catfish disease is caused by pathogen *Edwardsiella ictaluri*. It is one of the most serious diseases of catfish. The disease was first reported in 1976 (Hawke et al., 1981). Direct economic loss to the U.S. catfish industry due to ESC disease was estimated to be approximately $60 million annually. The disease affects all size classes of catfish including market size fish, and causes high mortality. About 19.3% of fingerling catfish and 36.6% of adult catfish had ESC disease problem as revealed by a survey in 2010 (APHIS, USDA).

Enteric septicemia of catfish outbreaks occur when water temperature is between 20 and 30°C. The symptoms of ESC include red and white ulcers; red spots under their heads or belly regions; and red pimples at the cranial foramen between the eyes that can from a “hole-in-head” condition (Hawke et al., 1998).

### Vaccine for ESC Disease Control

Vaccines for ESC have been researched for over three decades (Saeed and Plumb, 1986; Newman, 1993; Thune et al., 1994). The modified live *E. ictaluri* isolate RE-33 was the most successful vaccine. This isolate is not pathogenic but stimulate protective immunity (Klesius and Shoemaker, 1999; Shoemaker et al., 1999). The vaccine was commercialized by Intervet, Inc. The vaccine can be delivered through immersion, and the product is marketed under AQUAVAC-ESC (Intervet, Inc., Millsboro, DE, United States). The immersion studies reported successful protection in channel catfish fingerlings (Wise et al., 2000), and eggs (Shoemaker et al., 2002, 2007).

Additional studies for new vaccines for ESC disease are still being carried out. The *E. ictaluri* purA mutant (Lawrence et al., 1997), *E. ictaluri* ghost generated by PhiX174 lysis gene E (Wang et al., 2016), *E. ictaluri* outer membrane proteins (Yang Q. et al., 2016), ESC-NDKL1 generated by modification of FRDA gene in a gcvP-sdh mutant (Nho et al., 2017) and *E. ictaluri HemR* mutants (Abdelhamed et al., 2018) were constructed, and demonstrated to be potential vaccines against ESC in channel catfish. The immersion-based vaccines are typically used for mass delivery with very young fish whose immune system is not fully developed. An oral vaccine for channel catfish fingerlings was developed by attenuation of *E. ictaluri* isolate (S97-773). This oral vaccine increased antibody production by 18-fold, improved survival, and increased feed conversion efficiency and total harvest in pound trails (Wise et al., 2015). Additional evaluation confirmed that this vaccine could improve survival rate of channel catfish after exposed to *E. ictaluri* (Peterson et al., 2016).

The application and effectiveness of vaccine for ESC was also assessed by catfish producers. During 1989–1990, no obvious effects were observed after ESC vaccination. With the development of vaccine-oil emulsion coating method in 1990–1991, the overall survival rate in vaccinated fish was significantly improved (Thune et al., 1994). A survey indicated that 12.3% of the industry catfish fry was vaccinated against ESC, and 6.7% vaccinated catfish was used by the food-sized catfish producers during 2009 (Bebak and Wagner, 2012). About 41.9% of the producers that stocked ESC-vaccinated catfish believe that survival rate was improved in vaccinated fish. However, 37.5% of producers did not know whether vaccination changes survival rates (Bebak and Wagner, 2012). Apparently, ESC vaccines were quite effective in laboratories but were not consistently effective under the commercial pond environment (Dunham and Elaswad, 2018). Therefore, future studies are required to demonstrate the effectiveness of vaccines in commercial settings.

### Pathogenesis of *E. ictaluri*

The first step for *E. ictaluri* pathogenesis is interacting with potential susceptible cells, crossing mucosal barriers, and entering the host. The *E. ictaluri* infects channel catfish by utilizing host cellular transport systems to traverse through the epithelial-lined tissues, such as intestine and olfactory mucosa (Skirpstunas and Baldwin, 2002). The intestinal epithelial cells uptake *E. ictaluri* with the involvement of actin polymerization and endocytosis mediated by receptors (Skirpstunas and Baldwin, 2002). An RNA-Seq analysis of channel catfish intestinal immune response after *E. ictaluri* infection provided additional evidence for the involvement of the actin cytoskeletal polymerization remodeling in *E. ictaluri* infection (Li et al., 2012). Pathogenic members of the Enterobacteriaceae are commonly using receptor mediated endocytosis for entering host cells (Finlay and Falkow, 1989; Finlay et al., 1989). As an important member of Enterobacteriaceae, the infection mechanisms utilized by *E. ictalrui* are speculated to be similar: Cytochalasin D binds to actin resulting in microfilament depolymerization, which lead to the cell morphology alteration and bacterial adherence and internalization (Ewanowich and Peppler, 1990; Skirpstunas and Baldwin, 2002). The oligo-polysaccharide (OP-S) subunits of the bacterium *E. ictaluri* play a major role during pathogenesis (Santander et al., 2014). Recently, it was reported that NCK1 may play a key role in the *E. ictaluri* pathogenesis by initiating actin pedestal formation, and linking the surface located ligand and host cell receptors (Zhou et al., 2017b).

Once passing the epithelial barrier, the bacterium was believed to be taken up by propria macrophages (Miyazaki and Plumb, 1985). Normally, macrophages have a crucial role in resisting bacterial infections by migrating to the infection site, engulfing and killing pathogens (Kordon et al., 2018). However, numerous intracellular *E. ictaluri* were observed within clear vacuoles in the infected macrophage cultures of catfish (Booth et al., 2006), indicating that *E. ictaluri* could survive in channel catfish macrophages. The *E. ictaluri* in macrophages is then spread systemically through the bloodstream.

### Variation of ESC Disease Resistance of Catfish

Enteric septicemia of catfish disease resistance of catfish is highly variable among species and strains from different geographic locations (Dunham and Elaswad, 2018). In general, blue catfish is more tolerant to ESC than channel catfish, although significant difference in channel catfish ESC resistance was found among various strains and families (Wolters and Johnson, 1994; Wolters et al., 1996; Tucker and Hargreaves, 2004). Red river strain channel catfish showed highest ESC resistance, followed by Mississippi-select and Mississippi-normal fish (Wolters and Johnson, 1994). Hybrids of Norris female channel catfish and male blue catfish had intermediate resistance compared to blue catfish or channel catfish (Wolters et al., 1996; Hawke et al., 1998), while hybrids of NWAC103 channel catfish and blue catfish showed lower mortality than either parental species when exposed to natural diseases of ESC (Dunham et al., 2008), suggesting that combination of strains and families of the parent species influence disease resistance of the hybrids. Hybrid catfish have become common in the catfish production industry in the United States, now make up 50–70% of all catfish production (Dunham and Elaswad, 2018). Therefore, analysis of resistance among various hybrid catfish becomes important. In addition to production importance, the interspecific hybrid catfish offers an ideal system for the study of disease resistance by take advantage of the phenotypic contrast of channel catfish and blue catfish (Geng et al., 2015).

### Transcriptome Responses of Catfish After *E. ictaluri* Infection

Microarrays were initially used for the analysis of the transcriptome variance in channel catfish liver after the *E. ictaluri* infection. A set of genes were found to be differentially expressed after infection, particularly for the genes related to iron homeostasis (Peatman et al., 2007). Efforts were made to understand the molecular basis for disease resistance of blue catfish. Analysis of gene expression differences in liver of blue catfish after *E. ictaluri* infection by using microarrays indicated significant induction of inflammatory immune response pathways (Peatman et al., 2008). In particular, several MHC class I pathway members were differentially induced in blue catfish after *E. ictaluri* infection.

RNA-Seq was conducted to determine the involvement of the intestinal epithelium during *E. ictaluri* infection (Li et al., 2012). The results reveled that actin cytoskeletal polymerization, remodeling, and junctional regulation were important processes for *E. ictaluri* entry. A total of 1,633 genes were identified to be differentially expressed between *E. ictaluri* infected and control samples of channel catfish. After that, bulked segregant RNA-Seq (BSR-Seq) for the liver of hybrid catfish after *E. ictaluri* challenge identified a set of 1,255 differentially expressed genes between the resistant and susceptible catfish, and 8 linkage groups (LG) that contain QTL for ESC disease resistance (Wang et al., 2013). Many of the up-regulated genes in the susceptible hybrid catfish involve in the acute phase responses, as reported in the microarray analysis (Peatman et al., 2007, 2008). Similarly, RNA-Seq analysis of yellow catfish (*Pelteobagrus fulvidraco*) after *E. ictaluri* infection revealed a total of 5,527 differentially expressed genes, and the subsequent enrichment analysis revealed the involvement of innate and adaptive immune pathways (Zhu et al., 2017).

### QTL Analysis of ESC Disease Resistance

It was technically much easier to determine the genomic basis of ESC disease resistance after the publication of channel catfish reference genome sequence (Liu et al., 2016). Initially, a genome-wide association study (GWAS) was conducted with the fourth generation of backcross catfish progenies by using the 250K SNP array; a significant QTL on LG 1, and two suggestive QTL on LG 12 and 16 were identified to be associated with ESC disease resistance of catfish (Zhou et al., 2017b) (**Table 1**). A set of 16 genes with immune related functions were identified within the significant QTL on LG 1. Among them, the NCK1 gene was significantly upregulated in the intestine of channel catfish after *E. ictaluri* infection (Zhou et al., 2017a), and was considered as a possible candidate gene that confers ESC susceptibility, presumably by serving as an adaptor facilitating the pathogen entry into the host cells, similar to the situation of enteropathogenic *Escherichia coli* (EPEC) in humans (Zhou et al., 2017b).

**Table 1:** Summary of the QTL associated with ESC disease resistance of catfish as revealed by genome wide association studies.

Species	SNP array	Linkage group	Number of associated SNPs	Location	Significance	Reference
F4 backcross catfish	250K	1	5	32,103,802–32,547,486 bp	Significant	Zhou et al., 2017b
		12	6	24,072,309–24,210,072 bp	Suggestive	
		16	5	1,793,880–2,546,718 bp	Suggestive	
F2 backcross catfish	690K	1	45	33,233,001–33,712,554 bp	Significant	Tan et al., 2018
		23	3	7,793,018–8,136,119 bp	Significant	
Channel catfish	690K	1	10	5,948,310–9,638,510 bp	Significant	Shi et al., 2018
		26	3	1,639,485–1,666,617 bp	Significant	
		3	9	802,388–1,215,683 bp	Suggestive	
		21	2	3,278,086–3,284,725 bp	Suggestive	


Following that initial work, GWAS analysis was conducted in second generation of backcross catfish progenies by using the 690K SNP arrays (Zeng et al., 2017; Tan et al., 2018). Two QTL on LG 1 and LG 23 were identified to be significantly associated with ESC resistance (**Table 1**). Notably, the same genomic region on LG 1 was identified in both of the second and the fourth generation of backcross catfish progenies.

Most recently, GWAS analysis was conducted in channel catfish by using the 690K SNP array (Shi et al., 2018). Two QTL on LG 1 and one QTL on LG 26 were significantly associated with ESC resistance. Besides, three QTL located on LG 1, 3 and 21 respectively, were suggestively associated with ESC resistance (**Table 1**). Again, this study validated the QTL on LG 1 previously identified using the second and the fourth-generation backcross progeny populations. Taken together, it suggests that there are only a few major QTL associated with ESC disease resistance, making marker-assisted selection a possibility for genetic enhancement of ESC resistance.

### Gene Families Involved in ESC Disease Responses

RNA-Seq data were utilized to further investigate various gene families in channel catfish during *E. ictaluri* infection. Many gene families were induced after *E. ictaluri* infection, including heat shock proteins (Song et al., 2014, 2016; Xie et al., 2015), interlekin 17 (Wang X. et al., 2014), l-rhamnose-binding lectins (Thongda et al., 2014), nitric oxide synthase genes (Yao et al., 2014), claudins (Sun et al., 2015), peptidoglycan recognition proteins (Sun et al., 2014), Rab GTPases (Wang R. et al., 2014), Rho GTPase (Tan et al., 2017), suppressors of cytokine signaling genes (Yao et al., 2015), cytochrome P450 genes (Zhang et al., 2014), serpins (Li et al., 2015), bcl-2 genes (Yuan et al., 2016), cathepsins (Geng et al., 2015; Wang et al., 2015; Dong et al., 2016), tumor suppressor genes (Mu et al., 2015), complement regulatory protein genes (Jiang et al., 2015), septins (Fu et al., 2016), chemokines (Fu et al., 2017a,b,c), galectins (Zhou et al., 2016), apolipoproteins (Yang et al., 2017), receptor tyrosine kinases (Tian et al., 2015), NF-kB related genes (Wang et al., 2017c), phosphoinositide-3-kinases (Li et al., 2016), NCK and ABI adaptor genes (Zhou et al., 2017a), JAK and STAT genes (Jin et al., 2018). These studies indicated that most of the immune related genes were involved in the *E. ictaluri* disease responses. However, the regulatory network of immune system especially for those induced genes families is still unknown.

### Future Research of ESC Disease

Although research need to be done in multiple dimensions to understand and control ESC disease, the most promising ways are vaccine development and testing, and genetic enhancement of disease resistance. All studies of vaccines in the laboratory have shown their effectiveness and efficacy, but field studies, especially the timing, dose, and methods of vaccine application need to be determined for field conditions. Genetic studies of disease resistance indicated that only a limited number of QTL are involved in disease resistance against ESC disease. If validated, marker-assisted selection can be a quite effective approach for the enhancement of disease resistance. However, industry involvement is essential. With the known QTL information, it is now possible to determine the level of disease resistance of various lines used in the catfish industry. Introgression of the superior disease resistance QTL from blue catfish is a realistic approach to develop disease resistant catfish breeds.

## Part Ii: Columnaris Disease

### Impact of Columnaris Disease on Catfish Aquaculture

The columnaris was reported in 1922 from freshwater fish in Mississippi River (Davis, 1922), the threat of columnaris disease was one of the most significant risk to the aquaculture, particularly in the warm summer temperatures (Pacha, 1970; Becker and Fujihara, 1978). Although the pathogen *Flavobacterium columnare* is ubiquitous in the freshwater environment and can cause catastrophic mortalities in both wild as well as domesticated species (Schachte, 1983; Decostere et al., 1998; Morley and Lewis, 2010), channel catfish and blue catfish are especially vulnerable to *F. columnare* infections. The disease is often initiated as an external infection on the body surface, fins or gills, and subsequently developed into yellow-orange lesions along the dorsal midline, known as “saddleback” (Bullock et al., 1986; Plumb, 1999). In extreme conditions, the mortality for channel catfish fries after columnaris infection can reach up to 90%. The mortality rate for pond raised channel catfish can also reach as high as 50–60% (Decostere et al., 1998; Morley and Lewis, 2010; Plumb and Hanson, 2011), causing annual economic loss of approximately $30 million for the U.S. catfish industry (Shoemaker et al., 2011).

### Pathology and Host Specificity of Columnaris in Catfish

*Flavobacterium columnare* was originally named *Bacillus columnaris* and was renamed as *F. columnare* in Bernardet et al. (1996). It belongs to Flavobacteriaceae family; it is Gram negative, with smooth and yellowish colonies (Anacker and Ordal, 1955; Kunttu et al., 2009; Noga, 2011; Roberts, 2012). The *F. columnare* bacteria is rod shaped with 4–10 μm in length and 0.3–0.5 μm in width. *F. columnare* is featured with its rhizoid pattern on the low agar medium and its ability to grow on neomycin and polymixin B containing media (Griffin, 1992; Plumb, 1999). *F. columnare* can secret various of extracellular enzymes and is endowed with the ability to degrade polysaccharide, destructing gills, muscle and skin (Bernardet and Bowman, 2006). Based on restriction fragment length polymorphism (RFLP) analysis of the 16S rDNA, the *F. columnare* in North America can be assigned into three major categories (Wakabayashi, 1999). Among them, the genomovar I is more virulent in cold water species (Evenhuis and LaFrentz, 2016); genomovar II isolates are more lethal to the warm water species, such as catfish or bluegill (Triyanto, 1999; Arias et al., 2004; Darwish and Ismaiel, 2005; Olivares-Fuster et al., 2007; Shoemaker et al., 2008; Bullard et al., 2013; Mohammed and Arias, 2014). The pathogenesis of columnaris still remains largely unknown (Declercq et al., 2013). It is known that the adhesion ability, especially to the gills, is of vital importance to infection. The adhesion ability of *F. columnare* is affected by various factors such as the concentration of nitrite, organic matter or temperature (Decostere et al., 1999; Decostere et al., 2002). Thus, the outbreaks of columnaris disease is heavily dependent on the environmental factors such as the temperature, pH and hardness of water. The optimal temperature for the bacteria growth is 20–25°C (Bullock et al., 1986). In aquaculture productions, the risk for the columnaris is associated with the environmental stress; the risk increases with higher temperature, higher feeding rates, more organic loads as well as the increasing stocking densities (Olivares-Fuster, 2010). However, the decreasing of water hardness as well as the pH can suppress the spreading of *F. columnare* in the water (Fijan, 1968).

High levels of genetic variations exist among catfish with regard to their resistance against columnaris disease. For example, some channel catfish stocks are highly susceptible to columnaris infections such as the Rio Grande and Rio Grande S. stock (Dunham and Smitherman, 1984). Although channel catfish and blue catfish are both susceptible to *F. columnare* (Dunham et al., 1994), blue catfish is more susceptible to the disease in general. Hybrid catfish exhibit significant heterosis in terms of columnaris resistance (Birchler et al., 2003).

### Columnaris Vaccines for Catfish

Large efforts have been devoted to developing columnaris vaccines. Immersion with formalin-killed bacteria of channel catfish can provide a low level of protection from columnaris (Moore et al., 1990). Fryvacc 1 and Fryvacc 2 vaccines were developed and licensed for the control of *F. columnaris* (Bondad-Reantaso et al., 2012). The AQUAVAC-COL vaccine, developed from genomovar I and launched in 2005 (Arias et al., 2004), was demonstrated to be safe and effective to protect catfish from the infection of columnaris (Shoemaker et al., 2005, 2006, 2007, 2011). In addition, the AQUAVAC-COL can also protect other species such as largemouth bass from columnaris (Bebak et al., 2009). Gene expression analysis indicated that treatment with AQUAVAC-COL vaccine triggered expression of genes with the functions in cell maintenance of the host, immune responses, signal transduction and transcriptional regulation (Pridgeon and Klesius, 2010). However, AQUAVAC-COL is ineffective against genomovar II of columnaris. Most recently, a new vaccine (17-23), derived from attenuated genomovar II, was developed (Olivares-Fuster and Arias, 2011; Mohammed et al., 2013). RNA-seq analysis indicated that this vaccine can enhance the mucus system of fish and stimulate eosinophilic granular cells and the expression of neuroendocrine cells mediators in the fish gill (Zhang et al., 2017).

### Molecular Mechanisms of Columnaris Resistance

RNA-Seq analysis after *F. columnare* infection was conducted in catfish to identify transcriptome level of responses after infection. Since the gill is the primary organ for *F. columnare* adhesion and entry into the host (Decostere et al., 1999; Olivares-Fuster et al., 2011), RNA-Seq analysis of gene expression profiles was mostly conducted in gill tissues. Among the induced genes, rhamnose-binding lectin was significantly induced, along with down regulation of NF-κB signaling pathway (Sun et al., 2012). Previous researches had indicated that rhamnose-binding lectin has important role in the innate immune response (Watanabe et al., 2009). It functions as germline-encoded pattern recognition proteins by recognizing the lipopolysaccharides and lipoteichoic acid on the surface of Gram-negative and Gram-positive bacteria, respectively (Shiina et al., 2002; Tateno et al., 2002). The regulation of rhamnose-binding lectin during *F. columnare* infection suggested its potential role in bacterial aggregation and attachment (Beck et al., 2012). In addition, the comparisons between susceptible and resistance channel catfish transcriptome allowed the identification of the potential candidate genes associated with disease resistance. Some of the differentially expressed genes included inducible nitric oxide synthase 2b, lysozyme C, interleukin-8, and tumor necrosis factor-alpha, all of which were higher expressed in tolerant catfish than that in intolerant catfish. On the contrary, rhamnose-binding lectin was found to be induced in susceptible catfish than in resistant catfish (Peatman et al., 2013), suggesting association of high expression of rhamnose-binding lectin with susceptibility.

Genome-wide association study was conducted using backcross hybrid catfish (Geng et al., 2015). A 620-Kb region on LG 7 was observed to be significantly associated with columnaris resistance. Moreover, three additional QTL were identified on LG 7, 12 and 14. Interestingly, analysis of genes within the above identified QTL regions involve genes related to phosphoinositide 3-kinase pathway such as PIK3R3b, CYLD-like, ADCYAP1R1, ADCYAP1R1-like, and MAST2. Previous research indicated that phosphoinositide 3-kinase pathway are actively involved in the immune response, and 8 of 14 phosphoinositide 3-kinase genes were regulated after *F. columnare* infections in channel catfish (Li et al., 2016), suggesting the importance of phosphoinositide 3-kinase pathway for disease resistance of catfish against columnaris infections.

## Part Iii: *Aeromonas* Disease

### An Emerging Disease but Serious Threats to the Catfish Industry

Motile aeromonad septicemia (MAS), caused by the Gram-negative bacterium *Aeromonas hydrophila*, is an emerging disease threatening the catfish industry (Pridgeon and Klesius, 2011; Hossain et al., 2014; Pang et al., 2015). *A. hydrophila* is a mesophilic, rod-shaped, Gram-negative bacterium. The pathogen is able to infect a large number of hosts including amphibians, reptiles, avians, and mammals (Krieg and Holt, 1984), especially fish species (Ventura and Grizzle, 1987). Various fish species can be infected by *A. hydrophila*, including eel (*Anguilla anguilla*) (Esteve et al., 1994), Nile tilapia (*Oreochromis niloticus*) (Abd-El-Rhman, 2009), goldfish (*Carassius auratus*) (Harikrishnan et al., 2009), common carp (*Cyprinus carpio*) (Harikrishnan et al., 2003; Jeney et al., 2009; Robinson et al., 2012), and channel catfish (Hemstreet, 2010; Pridgeon and Klesius, 2011). MAS has been reported to cause huge economic losses in aquaculture. For instance, the cyprinid fish industry in central China was seriously impacted by MAS since 1989 (Zhang et al., 2014). The disease was first reported in the southern United States in the catfish industry during the summer of 2009, causing the loss of millions of pounds of food-size channel catfish (Hemstreet, 2010). The MAS outbreak was etiologically determined to be caused by a new virulent strain of *A. hydrophila* (Tekedar et al., 2013). Since the initial outbreak in 2009, the virulent *A. hydrophila* has spread to other states, including Mississippi and Arkansas (Pridgeon and Klesius, 2011), leading to an estimated loss of at least 12 million U.S. dollars (Hossain et al., 2014).

### Pathogenesis of *A. hydrophila*

The symptoms of *A. hydrophila* infection include sores on the skin, dropsy, necrosis, ulceration, and hemorrhagic septicemia (Pridgeon and Klesius, 2011). The pathogenicity of *A. hydrophila* is multifactorial (Pang et al., 2015). Many virulence factors have been identified, including secretion systems, motility and adhesins, toxins, enzymes, quorum systems, iron acquisition and antibiotic resistance (Tomás, 2012). The genomic sequence of *A. hydrophila* provided an insight into the pathogenicity (Pang et al., 2015). Pang et al. (2015) sequenced genomes of *A. hydrophila* strains and performed comparative genomic analyses. They found that utilization pathways for myo-inositol, L-fucose and sialic acid were associated with the virulent nature of epidemic *A. hydrophila*.

### Resistance of Catfish Against *Aeromonas* Disease

Similar to the performance in resistance to ESC and columnaris diseases, catfish exhibit a high level of variations in resistance against the MAS disease. Such variations are both interspecific and intraspecific between and within channel catfish and blue catfish. Blue catfish is observed to be more resistant to virulent *A. hydrophila* than either channel catfish or channel catfish × blue catfish hybrid catfish (Li et al., 2013). Moreover, the hybrid catfish was observed to be more resistant than channel catfish to *A. hydrophila* (Dunham and Masser, 2012). However, detailed analysis of variations among various channel catfish strains and blue catfish strains have not yet been conducted.

### Molecular Mechanisms Under *Aeromonas* Disease Resistance

Transcriptomic analyses following infection with *A. hydrophila* have been conducted in channel catfish and blue catfish. In one experiment, 60K Agilent microarrays were utilized to determine gene expression profiles in the skin of channel catfish and blue catfish after infection with virulent *A. hydrophila* (Li et al., 2013). In channel catfish, a total of 2,168 genes were significantly regulated after the bacterial challenge. These differentially expressed genes were involved in various pathways including immune, antioxidant, cytoskeletal, and nervous system pathways. Similarly, a set of 1,155 genes were differentially expressed in blue catfish after *A. hydrophila* infection. Dysregulation of genes was reported for genes involved in immunity, antioxidant responses, apoptosis, and cytoskeletal rearrangement. In addition to analysis in catfish, analysis of differently expressed genes in grass carp (*Ctenopharyngodon idellus*) infected with *A. hydrophila* revealed that they were implicated in complement system, cell adhesion molecules, apoptosis, and antioxidant responses. The similar functional pathways in different fish species after the same bacterial infection suggest evolutionary conservation of the molecular mechanisms of host response, and perhaps disease resistance (Yang Y. et al., 2016).

MicroRNAs (miRNAs) play significant roles in gene regulation by binding to complementary regions of mRNA to induce degradation or repress translation (Bartel, 2004). The identification of conserved and novel miRNAs was reported in catfish (Barozai, 2012; Xu et al., 2013), while the differential expression analysis of miRNAs after *A. hydrophila* infection has not been performed. Such analyses have been performed in other teleost fish species, contributing to a better understanding of the functional role of miRNAs in immune responses. For instance, 21 and nine miRNAs were found to be differentially expressed between *A. hydrophila*-susceptible and *A. hydrophila-*resistant grass carp in spleen and kidney, respectively (Xu et al., 2014, 2015). Results showed that miRNA targets were enriched in various physiological processes including immune system process.

### QTL for *Aeromonas* Disease Resistance

Identification of QTL and markers associated with disease resistance is of great significance because such information can be used for genetic improvement of broodstocks through breeding. In rohu (*Labeo rohita*), 21 SNPs on 10 LG were reported to be associated with the timing of death after *A. hydrophila* infection (Robinson et al., 2014). Genes of interest were identified that may be related to the observed QTL, including heat shock proteins, mucin, lectin, major histocompatibility loci, complement protein components, perforin, ubiquitin, T-cell antigen receptor and lymphocyte specific protein tyrosine kinase. To select for improved resistance to *A. hydrophila*, immune parameters were measured in resistant and susceptible lines of rohu to identify associated immune markers. The expression levels of ceruloplasmin and serum ceruloplasmin were found to be significantly higher in the resistant line over the susceptible line, suggesting the possibility of using immune parameters as indirect selection markers for disease resistance. In our laboratory, research for the identification of *Aeromonas* disease resistance QTL in catfish is underway, but unfortunately, grant funds are lacking to continue this important project.

## Part Iv: Heat Stress

### The Effects of Heat Stress on Catfish Industry

Stress caused by sudden changes in temperature or chronic heat stimuli above optimum conditions can interrupt cellular homeostasis and result in serious deficiency in development and growth, and even death. The global climate change is believed to affect all organisms in the ecosystems (Pörtner and Peck, 2010). In the freshwater systems, climate change is likely to increase water temperature, decrease dissolved oxygen and increase toxicity of pollutant (Ficke et al., 2007). Therefore, for the fish living in natural or artificial systems, global climate change will become a more significant stressor as it worsens over time. As a major factor of seasonal environment, water temperature experiences daily fluctuations and erratic changes. Physiologically, aquatic ectotherms has evolved to adapt to a limited scope of environmental variation, and living over that scope can be stressful or fatal (Roessig et al., 2004). The magnitude and direction of the temperature fluctuation, frequency of temperature change can significantly affect their life history (Todd et al., 2008). A variety of physiological functions such as growth, metabolism, reproduction success, food consumption, and the capacity to maintain internal homeostasis capacity of aquatic species will be affected in response to temperature fluctuation (Pörtner and Peck, 2010).

Catfish species have a wide range of natural habitats, and harbor great plasticity when they encounter temperature variations. Water temperatures in aquaculture ponds currently approach upper thermal tolerance levels for channel catfish, particularly in June-August, which routinely see daily maximum values of 29°C and higher (Arnold et al., 2013). Production rate in catfish ponds could be decreased due to reduced dissolved oxygen levels and an increased virulence of pathogens caused by high temperature. A drop in dissolved oxygen levels can lower the management capacity of water from uneaten feed, fecal matter, and fish metabolism, which could lower the reproductive capacity of catfish ponds (Ficke, 2005).

Heat stress also adversely affect growth, reproduction, and survival of channel catfish (McCauley and Beitinger, 1992; Arnold et al., 2013; Stewart et al., 2015). Three cycling upper-range temperature regimes (23–27°C, 27–31°C, and 31–35°C) of aquaculture environments affected the growth, and feeding performance of juvenile channel catfish (Arnold et al., 2013). The survival rate of catfish was significantly decreased for individuals under the highest temperature regime. Also, the catfish growth rate was decreased. Therefore, increased water temperature may present major challenges to the catfish industry (Arnold et al., 2013).

### Heat Tolerance Between Channel Catfish, Blue Catfish and Hybrid Catfish

In United States, the majority of catfish production are in the southeast (92%), where some of the warmest temperatures are found (Stewart et al., 2015). Channel catfish has a natural distribution from southern Canada to northern Mexico, which represents a temperature range from 5 to 35°C (Bennett et al., 1998; Tavares-Dias and Moraes, 2007; Stewart et al., 2015). Blue Catfish are distributed further south, spanning from the Mississippi River basin and Gulf Coast through Mexico and into Guatemala and Belize (Graham, 1999). In general, blue catfish has a higher capability of heat tolerance than channel catfish. The hybrid catfish also have a higher level of heat tolerance than channel catfish (Stewart et al., 2015). It was reported that the optimum water temperature for channel catfish best growth performance ranging from 27 to 32°C (Stewart et al., 2015). However, the Southeastern U.S. ponds reach daily maximum as high as of 34–36°C with daily fluctuations averaging 4°C in May–August (Arnold et al., 2013), indicating that aquacultured catfish is under constant heat stress.

Knowledge about the heat tolerance between different channel catfish strains, blue catfish strains and their hybrid catfish is very limited. It was first found that little to no geographic variation in incipient upper lethal temperature (IULT) of Channel Catfish from Florida and Ohio (Hart, 1952). However, the sample size for this study was small. Thereafter, critical thermal maximum (CTmax) was used to examine the thermal sensitivity of catfish to acute temperature fluctuations (Bennett et al., 1998). It was observed that the CTmax ranged from 38.6°C to 40.3°C for two geographically distinct strains of channel catfish. Catfish with a southern distribution (Delta Select strain, from the Mississippi Delta, MS, United States) showed a higher CTmax than catfish with a northern distribution (Red River strain, from the Red River, ND, United States) (Stewart and Allen, 2014). In hybrid catfish, geographic differences in thermal tolerance were observed as well, providing an evidence of genetic component for thermal tolerance in catfish.

### The Molecular Mechanisms Underlying Heat Stress in Catfish

Research using several model species revealed that a series of evolutionarily conserved stress-associated genes display distinct expression for heat stress, including genes involved protein folding and repair, protein degradation and biosynthesis, energy metabolism, cell cycle and signaling, cytoskeletal reorganization and apoptosis (Kültz, 2005; Logan and Somero, 2011; Liu et al., 2013). Increasing the levels and magnitudes of stress sequentially can lead to different components of the stress response (Feder and Hofmann, 1999; Kültz, 2005; Logan and Somero, 2011; Logan and Buckley, 2015). For instance, under mild heat stress, chaperone proteins could be highly expressed to refold proteins to maintain protein homeostasis (Feder and Hofmann, 1999). Under moderate levels of heat stress, many abnormal folded proteins will be degraded by proteolysis processes. Additionally, under sever heat stress conditions, basal cell activities may be induced because of the cellular damage. Such as the cell proliferation and cytoskeletal reorganization may be up-regulated, which has attendant effects on cellular structure and function. Furthermore, when suffering severe heat stress, significant cell damage will lead to induction of apoptotic pathways (Buckley et al., 2006; Logan and Somero, 2011).

The first described proteins in relation to heat stimuli are heat shock proteins (HSPs), which are a class of proteins that are produced by cells in response to environmental stresses (Zhu et al., 2016). Several heat shock proteins are play function as intra-cellular chaperons to fold and unfold other proteins under heat stress, oxidative and other cellular stimuli (Buckley and Hofmann, 2004). In catfish, some of heat shock genes were first characterized in response to heat. The heat-shock protein (stress-70 family) was isolated from liver of channel catfish in Abukhalaf et al. (1994). Stress-70s from tissues of several fish species contain common antigenic determinants of the protein (Abukhalaf et al., 1994). It has been suggested that expression levels or accumulation of this protein could be a useful predictor of a stressful environment. Increased expression of stress-70s were detected in stressed fish (Welch, 1990). Straight after, the cDNA sequence of a member of heat shock protein 70 family in channel catfish was identified in 1996. The CF Hsp70 mRNA expression in three leukocyte cell lines was examined as well (Luft et al., 1996). At optimal culture temperature (27°C), the CF Hsp70 mRNA was constitutively expressed at high levels. However, at heat shock condition (37°C), only a modest up-regulation of CF Hsp70 expression was detected (Luft et al., 1996).

In order to obtain a broad understanding of gene expression profiles under heat stress in catfish, RNA-Seq was conducted in hybrid catfish (Liu et al., 2013). In this study, samples of gill and liver from heat sensitive, tolerant and control catfish groups were used for conducting RNA-Seq. In total, 2,260 differentially expressed genes were identified between control fish and heat sensitive and/or heat resistant fish following heat stress. The differentially expressed genes were classified into six functional groups, including protein folding, protein degradation, protein biosynthesis, energy metabolism, molecule and ion transport, and cytoskeleton reorganization (Liu et al., 2013). Especially, the dramatically up-regulated genes are those participated in protein folding and degradation, oxygen transport, and metabolic process. Whereas, the lethal heat stress (37°C) in this study caused significant reduction of expression levels of genes encoding for general protein synthesis. Gene encoding for molecular chaperones were most strongly up-regulated. The chaperones are critical in stabilizing protein homeostasis under heat stress in order to avoid aggregation and degradation of proteins (Parsell and Lindquist, 1993). For example, members of the HSP40 family (DNAJA1, DNAJA4, and DNAJB1B), HSP70 proteins (HSPA5, HSPA1A, and HSC73L), and the HSP90 family (HSP90AA1, HSP90AA2, HSP90AB1, and HSP90B1) were significantly upregulated after heat challenge. In spite of the protein folding and rescue process participated by chaperone proteins and related factors, some damaged proteins that cannot be rescued by chaperones are degraded by either autophagy-lysosomal pathway or ubiquitin proteasome pathway (UPP). Numerous proteases were significantly induced in this study, such as cathepsins and legumain. In comparison, heat stress caused reduced expression levels for several genes involved in UPP proteolysis process, demonstrating the downregulation of the ATP-dependent proteolysis way (Liu et al., 2013). Genes with functions in transporting lipids, proteins, iron and glucose were identified after heat stress in catfish (Liu et al., 2013). As expected, the expression of genes involved in regulating metabolism and repair system showed up regulation in response to heat, because these processes are energy-costing. However, several genes in relation to respiratory chain were down-regulated, such as genes coding for mt-ND1, mt-ND2, mt-ND6, and COX2. It’s well recognized that heat stress preferentially leads to upregulation of specific stress-related genes while downregulation of general genes involved in protein synthesis (Buckley and Hofmann, 2002; Buckley et al., 2006). Similarly, our study observed significantly reduction of ribosomal protein genes in gills after heat stress stimuli as well. In addition to affecting the internal cellular activities, heat stress can induce the expression of several cytoskeleton-associated proteins, including G3BP2, TPM4, MMP9, MMP13, MMP18, COLLA1A, and COLLA1B.

### QTL Associated With Heat Stress in Catfish

Genome-wide association study analysis has been conducted to detect QTL for heat stress tolerance. With a well-assembled reference genome sequence, potential candidate genes within QTLs can be identified simultaneously, which is a useful way to understand the molecular mechanisms of a trait by identification of genes in proximity to QTL (Dikmen et al., 2013; Geng et al., 2015). As described above, GWAS was conducted to identify QTL associated with several important traits, including disease resistance for columnaris (Geng et al., 2015) and ESC (Zhou et al., 2017b; Shi et al., 2018; Tan et al., 2018), growth rate (Li et al., 2017), head size (Geng et al., 2016), body conformation (Geng et al., 2017), and low oxygen tolerance (Wang et al., 2017b; Zhong et al., 2017). As the global climate change worsens over time, it is important to develop heat-tolerant lines. GWAS was conducted to identify heat stress-associated QTL, by using interspecific backcross progenies and the 250K catfish SNP array (Jin et al., 2017).

At the genome-wide significance level, three SNP markers were found to be associated with heat stress. One SNP was identified from LG 14, two additional SNPs were found from LG 16 (Jin et al., 2017). Around the three heat-related SNP markers, 14 heat stress associated genes were found. Among them, five genes (TRAF2, FBXW5, ANAPC2, UBR1 and KLHL29) are involved in the proteolysis process via the way of ubiquitination (Jin et al., 2017). This observation indicated that such lethal heat treatment resulted in irregular folding of, and irreversible disruption to, proteins, therefore, increased proteolytic degradation were required to remove the damaged proteins. However, observations from RNA-Seq analysis indicated that molecular mechanisms during heat stress is very complex; the levels and magnitudes of stress can lead to different components of the stress responses (Logan and Buckley, 2015). In the GWAS study, genes involved in protein biosynthesis, protein folding, molecule and iron transport, cytoskeletal reorganization and energy metabolism processes were also identified in the genome-wide significantly associated regions (Jin et al., 2017). The results further supported the notion that except for the involvement of intensive proteolysis, heat stress induces various molecular mechanisms to maintain cellular hemostasis (Liu et al., 2013; Jin et al., 2017).

### Comparison of Molecular Responses of Catfish to Diseases and Heat Stress

The heat-shock response (HSR) is one of the highly conserved molecular responses to disruptions of protein homeostasis. HSPs are induced to protect the proteome from elevated temperatures. As mentioned above, HSPs are significantly induced under acute and mild heat stresses. Likewise, in response to acute stress from pathogen infection and hypoxia, HSPs are also induced. Because of the constitutive expression in non-stressed cells, HSPs are always been used as housekeeping proteins (Song et al., 2014). However, recent studies have revealed that several HSPs play essential roles in innate and adaptive immunity. For instance, HSPs play roles in regulate humoral immune responses; in extracellular environment, HSPs are considered to be a danger signal to trigger innate immune cells such as macrophages and dendritic cells (Srivastava, 2002). In catfish, families of HSP40, HSP60, HSP70, HSP90 and sHSP were characterized, and their expression profiles following *E. ictaluri* and *F. columnare* bacterial infection were determined. Pathogen-specific, tissue-specific and time-specific patterns were found of these heat shock genes after infection with the two diseases (Song et al., 2014, 2016; Xie et al., 2015). Most of the HSP genes were induced at the earliest stages of disease infections, while more and more HSP genes became down-regulated with the progression of the diseases.

Similarly, the up-regulation of claudin and cathepsin genes were detected after bacterial infection and heat stimuli by RNA-Seq studies. As a group of transmembrane proteins, claudins have significant functions in cytoskeletal reorganization (Sun et al., 2015). Claudin-1, a, e, i, were significantly induced after heat stress in catfish (Liu et al., 2013). Using existing RNA-Seq datasets, Sun et al. (2015) reported dramatic down-regulation of claudins at the earliest phase (3 h) of infection, suggesting that pathogens could interrupt the mucosal barrier via repressing activities of claudins (Sun et al., 2015). Cathepsins are a large group of proteases that serve as enzymes degrading damaged proteins to avoid forming cytotoxic aggregates. After heat stress, the expression of cathepsin Z, B, D, L were dramatically induced (Liu et al., 2013). After infection of *E. ictaluri* and *F. columnare*, expression of catfish cathepsin D, H, L, S genes were significantly induced. However, these genes displayed pathogen-specific or tissue specific patterns following infections, indicating their disparate roles or distinct tissue-specific functions in immunity (Yeh and Klesius, 2009; Feng et al., 2011; Wang et al., 2015; Dong et al., 2016). However, further functional studies are required to fully understand the roles of these genes after biotic and abiotic stresses.

## Part V: Hypoxia

### Impact of Hypoxia on the Catfish Industry

Dissolved oxygen is important for the survival and life of aquatic organisms. Under aquaculture conditions, hypoxia can be caused by natural phenomena (e.g., weather, temperature, or water flow rate), water pollution and eutrophication, high stocking density, and improper use of aeration (Wu, 2002; Zhang et al., 2010; Green et al., 2016). In spite of the high tolerance of catfish to low levels of oxygen, hypoxia can still lead to huge economic losses to the catfish industry. Exposure to low oxygen stress can affect growth rate (Mallya, 2007), feed conversion rate (Buentello et al., 2000), and increase the susceptibility to infectious diseases of catfish (Geng et al., 2014). Further, hypoxia can also negatively affects several additional aspects of biological behaviors such as increased vulnerability to predation (Domenici et al., 2007), reduced reproductive capacity (Landry et al., 2007), delayed embryonic development (Shang and Wu, 2004), and reduced metabolic rate (Richards, 2009).

### Variation of Hypoxia Tolerance Among Catfish Species and Strains

In general, channel catfish has been believed to be more tolerant to low oxygen than blue catfish (Graham, 1999). In one experiment, the tolerance to low dissolved oxygen of six strains in channel catfish, including 103KS strain, Kansas strain, KMix strain, Marion strain, Marion S strain, and Thompson strain, was evaluated (Wang et al., 2017a). Compared with other channel catfish strains, two strains (103KS strain and Marion S strain) exhibited higher tolerance to low oxygen stress, whereas Marion strain of channel catfish showed the lowest tolerance to hypoxia stress (Wang et al., 2017a). Compared with their maternal parent (channel catfish) and paternal parent (blue catfish), interspecific hybrid catfish exhibited stronger tolerance to low dissolved oxygen levels (Dunham et al., 1983). The strains of their parents have an influence on the tolerance of hybrids to low dissolved oxygen (Dunham et al., 2014). For instance, at low temperatures (9 and 17°C), hybrid catfish sired by Alabama strains showed the highest tolerance to hypoxia stress than hybrid catfish sired by other strains of blue catfish (Dunham et al., 2014).

### Molecular Mechanism on Hypoxia Tolerance in Catfish

Fish live in aquatic environments with low dissolved oxygen levels. They have evolved with various strategies to adapt to such environment, including their enzymatic activities, metabolic rate, and increased surface (gill lamellae) for oxygen exchange (Burggren and Cameron, 1980; Jensen et al., 1993). HIF-1 and HIF-2 are two important regulators for hypoxia responses and oxygen homeostasis (Semenza, 2000; Giannetto et al., 2015). After hypoxia treatment, the HIF genes were differentially expressed in catfish at the transcriptional levels as well, rather than just at the post-translational levels as believed previously (Geng et al., 2014). Several gene families were involved in the hypoxia response including receptor tyrosine kinases (RTKs) (Tian et al., 2015), bcl-2 genes (Yuan et al., 2016), CC and CXC chemokine families (including ligands and receptors) (Fu et al., 2017a,b,c), and claudin genes (Sun et al., 2015).

Multiple molecular pathways are involved in hypoxia responses. These molecular pathways included HIF signaling pathway (Bruick, 2003), PI3K-Akt signaling pathway (Mazure et al., 1997), MAPK signaling pathway (Koong et al., 1994), mTOR signaling pathway (Lu et al., 2006), VEGF signaling pathway (Lee et al., 2007), AMPK signaling pathway (Nagata et al., 2003) and FoxO signaling pathway (Eijkelenboom and Burgering, 2013). In a recent study with catfish, Yang et al. (2018) used RNA-Seq analysis to provide systematic knowledge about gene regulation under hypoxia conditions. Through enrichment analysis of differentially expressed genes, they confirmed the involvement of HIF, MAPK, PI3K/Akt/mTOR and Ras signaling pathway under hypoxia.

### GWAS Studies on Low Oxygen Tolerance in Catfish

Two GWAS studies were recently conducted on hypoxia tolerance, using channel catfish (Wang et al., 2017b) and hybrid catfish (Zhong et al., 2017). In one experiment with six strains of channel catfish, QTL were identified in all six strains, but they rarely overlaped, suggesting the complexity of the genetic architecture of hypoxia tolerance in different channel catfish strains (Wang et al., 2017b). Genes annotated within QTL were involved in not only MAPK signaling pathway but also PI3K/AKT/mTOR signaling pathways (Wang et al., 2017b). It is apparent that mutations in any of the several gene pathways can lead to QTL associated with tolerance to low oxygen.

In another experiment, Zhong et al. (2017) used the catfish 250K SNP arrays to determine QTL with the hybrid catfish. A total of four QTL (on LG 2, 4, 23 and 29) involved in hypoxia tolerance were identified in hybrid catfish. In pathway analysis, annotated genes within QTL associated with hypoxia tolerance belonged to several pathways, for instance, PI3K/Akt/mTOR signaling pathway (Zhong et al., 2017). The QTL identified from the interspecific hybrid catfish, for the most part, had rare overlaps with the QTL within the six strains from channel catfish (Wang et al., 2017b), further supporting the notion that the genetic architecture of low oxygen tolerance in catfish is extremely complex (Zhong et al., 2017).

### Potential Interactions Between the Responses to Hypoxia Stress and Bacterial Infection in Catfish

The interdependence of hypoxic responses and innate immune responses has been observed in mammals (Nizet and Johnson, 2009). For instance, the key regulator of hypoxia responses, HIF-1α, has been demonstrated to function as an essential regulator of inflammation and innate immunity (Zinkernagel et al., 2007). Similarly, the potential linkage between immune responses and the responses to hypoxia were also observed in catfish. After infection with high challenge dose of *E. ictaluri*, channel catfish in the hypoxic groups had higher mortality (∼36%) than the catfish in control groups (∼12%) (Welker et al., 2007). Furthermore, PI3K signaling pathway was found to be involved in the molecular responses to both hypoxia stress and bacterial infections, as supported by GWAS analysis on tolerance to low dissolved oxygen of both channel catfish (Wang et al., 2017b) and hybrid catfish (Zhong et al., 2017), and the resistance to columnaris disease in hybrid catfish (Geng et al., 2015). In spite of being preliminary, these studies suggested similarities of responses to bacterial infections and those to hypoxia, and potential interactions of the host responses to biotic and abiotic stresses.

## Author Contributions

TZ, ZY, ST, YJ, YY, HS, WW, DN, LG, and WJ wrote the draft manuscript. DG and ZL reviewed, revised, and finalized the manuscript.

## Conflict of Interest Statement

The authors declare that the research was conducted in the absence of any commercial or financial relationships that could be construed as a potential conflict of interest.
